# *N* 1-Arylation of 1,4-Benzodiazepine-2-ones with Diaryliodonium Salts

**DOI:** 10.1055/s-0036-1590920

**Published:** 2017-09-25

**Authors:** Raysa Khan, Robert Felix, Paul D. Kemmitt, Simon J. Coles, Graham J. Tizzard, John Spencer

**Affiliations:** a Department of Chemistry, School of Life SciencesUniversity of Sussex, Falmer, BN19QJUKj.spencer@sussex.ac.uk; b Tocris, Bio TechneThe Watkins Building, Atlantic Road, Bristol, BS11 9QDUK; c IMED Oncology, AstraZeneca310 Cambridge Science Park, Milton Road, Cambridge CB4 0WGUK; d UK National Crystallography ServiceChemistry, University of Southampton, Highfield, Southampton, SO17 1BJUK

**Keywords:** benzodiazepines, N-arylation, iodonium salt, privileged scaffold, benzotriazepine

## Abstract

A library of *N* 1-arylated 5-phenyl-1,3-dihydro-2 *H* -1,4-benzodiazepin-2-ones has been synthesized starting with unsymmetrical diaryliodonium salts using aqueous ammonia as a base. This can also be applied to a similar 1,3,4-benzotriazepin-2-one derivative.

Compounds containing a 1,4-benzodiazepine scaffold are often termed as ‘privileged structures’ and are of significant interest to organic and medicinal chemists. 1 2 3 4 5 6 7 8 9 10 11 12 13 14 15 16 17 18 Many bioactive 1,4- benzodiazepines include *N* -arylated benzodiazepines; for example, the benzodiazepine derivative **A** (Figure 1 ) is a bradykinin antagonist 19 and the related benzotriazepine **B** is an antagonist at the parathyroid hormone (PTH)-1 receptor. 20 Typically *N* -arylated benzodiazepines can be prepared by transition-metal- catalysed couplings, often with copper, with various arylating agents. Generally, the reaction scope is limited with these routes and often requires high temperatures and strong bases. 19 ^,^ 21 22 23

**Figure 1:**
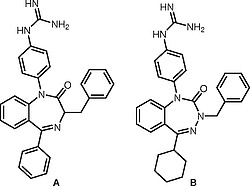
Bioactive *N* -arylated Benzodiazepine and Benzotriazepine

Being able to generate libraries of diverse analogues, in this case by adding *N* -functionality to a privileged core unit, using mild and efficient methodologies, can substantially improve SAR studies (structure–activity relationship) and optimise the drug development process potentially repurposing privileged scaffolds for new biological targets. 24 25

We have an active interest in benzodiazepines 26 27 and recently reported a method to functionalise 5-phenyl-1,3-dihydro- *2H* -1,4-benzodiazepin-2-ones via a late-stage palladacycle assisted *ortho* C–H activation protocol. 28 29 Herein we present our approach to generate a series of *N* 1-arylated 1,4-benzodiazepines using diaryliodonium salts. The latter react with nucleophiles in the absence of transition-metal catalysts and are commonly used in organic synthesis as electrophilic reagents. 30 31 32 33 34 35

Novak et al. recently reported a protocol for the *N* -arylation of pyrazoles. 36 A quick screen of conditions, adapting this protocol using diaryliodonium salts with weak bases under mild conditions, showed that it was indeed possible to perform similar arylations on the 1,4-benzodiazepine system. Upon initial screening of a number of solvents, 1,2-dichloroethane (DCE) was found to give the best results (Table 1 , entry 2). Solvents such as polypropylene glycol (PEG) and acetic acid (AcOH) gave poor yields. Similar results were observed on pyrazoles by Novak et al. where aprotic solvents, immiscible in water, produced the best results.

**Table:** **Table 1** Optimization of *N* -Arylation of 1,4-Benzodiazepines – Solvent Effects

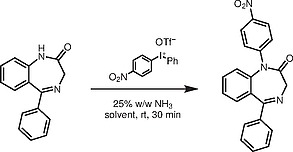		
Entry	Solvent	Conversion (%) ^a^
1	toluene	95
2	DCE	99
3	PEG	–
4	AcOH	–
5	CHCl _3_	85

^a^ LC–MS conversion.

A number of bases were tested subsequently and both NH _3_ (25% w/w) and NaOH (sat. aq.) gave similar and the best results (Table 2 , entries 1, 2).

**Table:** **Table 2** Optimization of *N* -Arylation of 1,4-Benzodiazepines – Base ­Effects

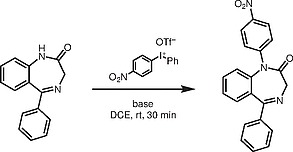		
Entry	Base	Conversion (%) ^a^
1	NaOH (sat. aq.)	99
2	NH _3_ (25% w/w)	99
3	K _2_ CO _3_	80
4	NaH	-

^a^ LC–MS conversion.


**Scheme 1** *N* -Arylated 1,4-Benzodiazepines


Hence, optimal conditions appeared to use NH _3_ (aq.), DCE at room temperature for 30 min. Next, a series of functionalized 1,4-benzodiazepines was *N* -arylated using (4-nitrophenyl)phenyliodonium triflate in good to excellent yields (Scheme 1 ). Generally, in transition-metal-free processes unsymmetrical diaryliodonium salts give a mixture of products where both groups are transferred and the transfer of more sterically hindered and electron-withdrawing groups is preferable. 34 However, in this case (Scheme 1 ) only the nitrophenyl group was transferred. We were able to *N* -arylate quite sterically hindered benzodiazepines such as **3e** , **3f** , and **3g** . Of note, **3e** is a key intermediate towards **A** . We were also pleased to be able to conduct *N* -arylation on a previously *ortho* -arylated hindered benzodiazepine, **3h** , in good yield, whose structure was also confirmed by X-ray crystallography. Such molecules may be useful precursors to, e.g., α-helical mimetics in medicinal chemistry. 37 38

The use of other unsymmetrical diaryliodonium triflates was also explored (Table 3 ), which required longer reaction time and led to both aryl groups being transferred to obtain **3i** – **l** . As expected, the transfer of more sterically hindered or less electron-rich groups was preferred. Further attempts to use unsymmetrical diaryliodonium salts such as phenyl(3-methylphenyl)iodonium triflate, phenyl(4-methylphenyl)iodonium triflate, and (2-methylphenyl)(2,4,6-trimethylphenyl)iodonium triflate gave little or no products. Additionally, attempted *N* -arylation with symmetrical diaryliodonium triflates or tetrafluoroborates such as bis(2-fluorophenyl)iodonium tetrafluoroborate and bis(4-bromophenyl)iodonium triftlate gave, at best, traces of products.

**Table:** **Table 3** Further *N* -Arylation of 1,4-Benzodiazepines ^a^

Salt	Product (major)	Product (minor)
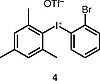	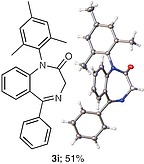	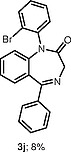
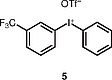	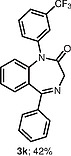	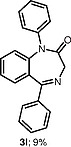

^a^ Reaction time = 8 h.

We have briefly explored the *N* -arylation on a 1,3,4-benzotriazepine **6** , which resulted in diarylation and yielded **7** (Scheme 2 ).


**Scheme 2** *N* -Arylation on a 1,3,4-Benzotriazepine


Interestingly, the iodonium salts were observed to undergo reaction with water present in the reaction to give diarylether products. The ether product is only observed in substantial amounts when the benzodiazepine substrates react poorly with the diaryliodonium salts (Table 4 ). The ether product **10** was also obtained merely by stirring the iodonium salt with water in DCE with a mild base for 20 min at room temperature with a yield of 43%. Olofsson et al. have reported the synthesis of related diarylethers by reacting diaryliodonium salts with phenols in the presence of mild bases. 39

**Table:** **Table 4** Diaryl Ether Formation

Substrate	Expected product	Observed product
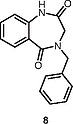	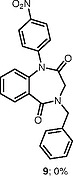	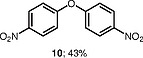
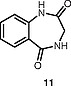	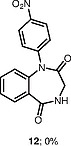	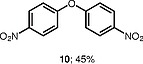

In summary we have presented a mild metal-free route to *N* -arylated benzodiazepines, three of which were structurally characterized in the solid state ( **3a** , **3h** , **3i** ). 40 41
