# Supplementary material for: N 1-Arylation of 1,4-Benzodiazepine-2-ones with Diaryliodonium Salts
Source: Synlett. 2017 Sep 25;29(2):193–8. doi: 10.1055/s-0036-1590920 (PMC6193364; doi:10.1055/s-0036-1590920)
Supplement: Supplementary file 1 — Supporting Information [file sup_st-2017-d0556-l_10-1055_s-0036-1590920.pdf]

Synlett
Accounts and Rapid Communications in Chemical Synthesis

Supporting Information
for DOI: 10.1055/s-0036-1590920
© Georg Thieme Verlag KG Stuttgart · New York 2017

Thieme

N-Arylation of 1,4-Benzodiazepines with Diaryliodonium Salts

Raysa Khan,[a] Robert Felix,[b] Paul D. Kemmitt,[c] Simon J. Coles,[d] Graham J. Tizzard, [d] and John Spencer*,[a]

[a] Department of Chemistry, School of Life Sciences, University of Sussex, Falmer, BN1 9QJ, UK. Email: j.spencer@sussex.ac.uk.

[b] Tocris Bioscience, Tocris House, IO Centre, Moorend Farm Avenue, Bristol, BS11 0QL, UK.

[c] Oncology, AstraZeneca, 310 Cambridge Science Park, Milton Road, Cambridge CB4 0WG, U.K.

[d] UK National Crystallography Service, School of Chemistry, University of Southampton, Highfield, Southampton, SO17 1BJ, UK.

Experimental Section

All reactions were conducted under an inert atmosphere unless specified otherwise. All commercially purchased materials and solvents were used without further purification unless specified otherwise.

NMR spectra were recorded on a Varian VNMRS 500 (^{1}H: 500 MHz, ^{13}C: 126 MHz) spectrometer and prepared in deuterated solvents such as $CDCl_3$ and DMSO-d_6. ^{1}H and ^{13}C chemical shifts were recorded in parts per million (ppm). Multiplicity of ^{1}H-NMR peaks are indicated by s – singlet, d – doublet, dd – doublets of doublets, t – triplet, pt – pseudo triplet, q – quartet, m – multiplet and coupling constants are given in Hertz (Hz).

Electrospray ionisation – high resolution mass spectra (ESI-HRMS) were obtained using a Bruker Daltonics Apex III where Apollo ESI was used as the ESI source. Most analyses were conducted by Dr A. K. Abdul-Sada at Sussex. The molecular ion peaks [M]+ were recorded as mass to charge (m/z) ratio. A portion of the high-resolution mass spectrometry (HRMS) measurements were also performed by the EPSRC National Mass Spectrometry Facility, University of Swansea.

LC-MS spectra were acquired using a Shimadzu LC-MS 2020, on a Gemini 5 µm C18 110 Å column and percentage purities were run over 30 minutes in water/acetonitrile with 0.1% formic acid (5 min at 5%, 5%-95% over 20 min, 5 min at 95%) with the UV detector at 254 nm. Purifications were performed by flash chromatography on silica gel columns or C18 columns using a Combi flash RF 75 PSI, ISCO unit.

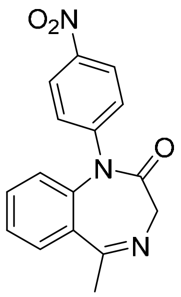
O2N
N
O
N

3a; 96%

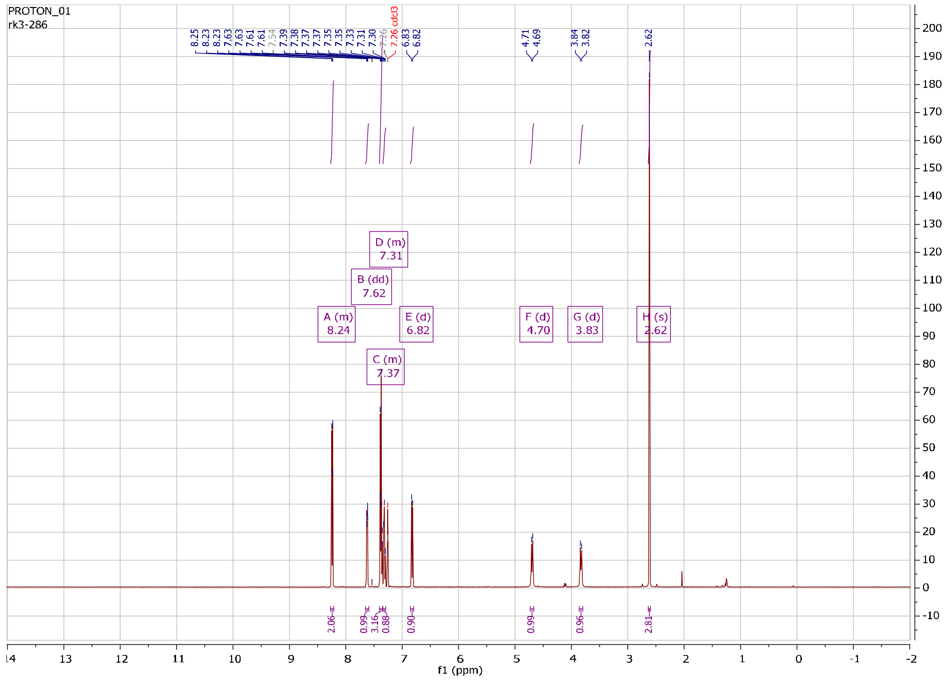
PROTON_01
rk3-286
8.25
8.23
8.23
7.63
7.61
7.61
7.54
7.39
7.38
7.37
7.37
7.35
7.35
7.33
7.31
7.30
7.26
7.26 cdcl3
6.83
6.82
4.71
4.69
3.84
3.82
2.62
D (m)
7.31
B (dd)
7.62
A (m)
8.24
E (d)
6.82
F (d)
4.70
G (d)
3.83
H (s)
2.62
C (m)
7.37
2.06
0.99
3.16
0.88
0.90
0.99
0.96
2.81
f1 (ppm)

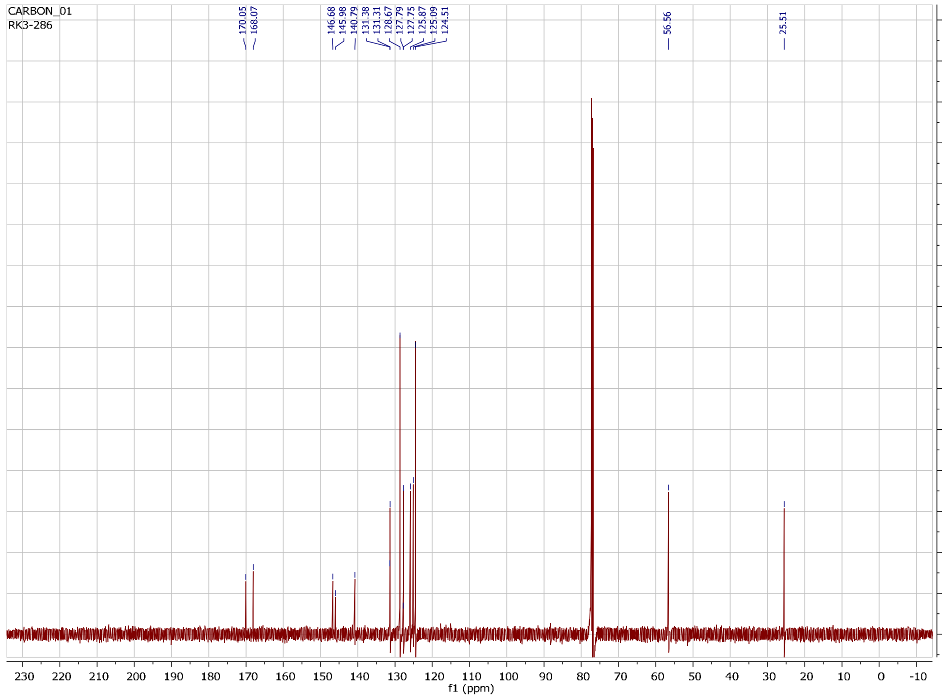
CARBON_01
RK3-286
170.05
168.07
146.68
145.98
140.79
131.38
131.31
128.67
127.79
127.75
125.87
125.09
124.51
56.56
25.51
f1 (ppm)

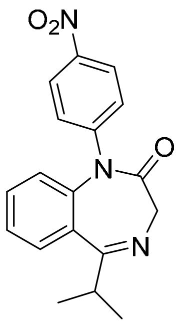

3b; Quantitative

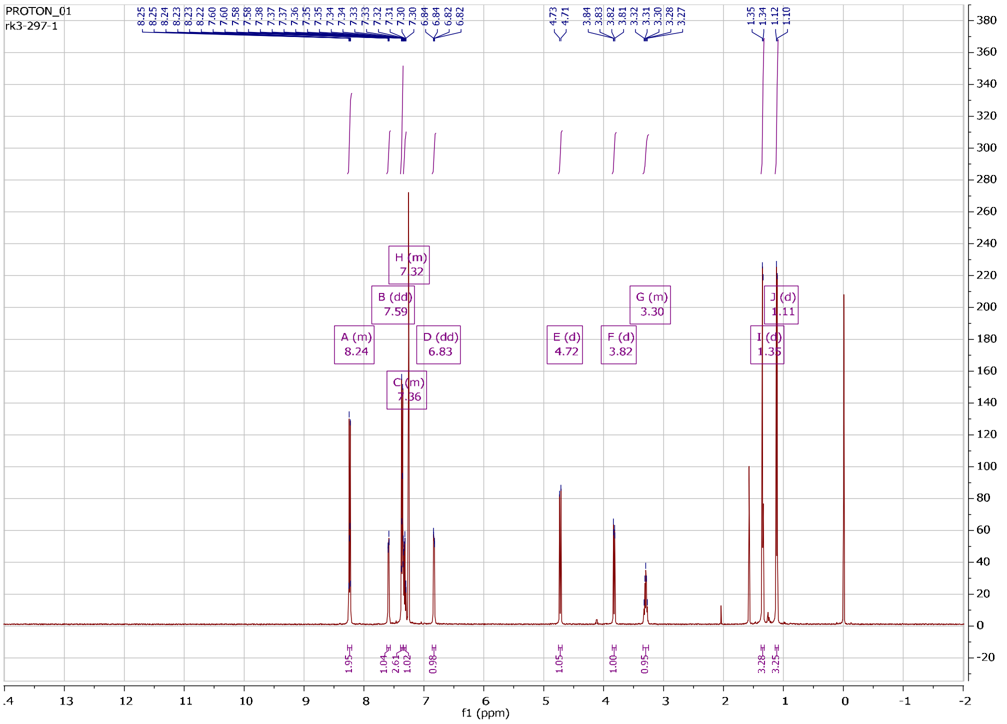

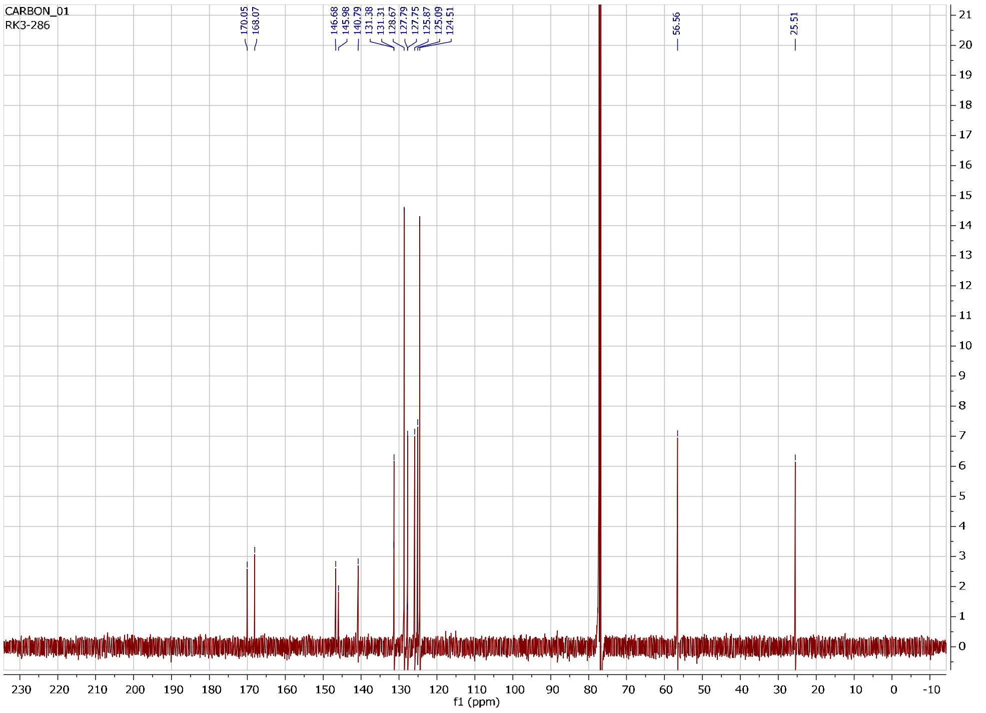

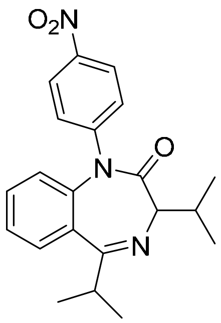

3c; Quantitative

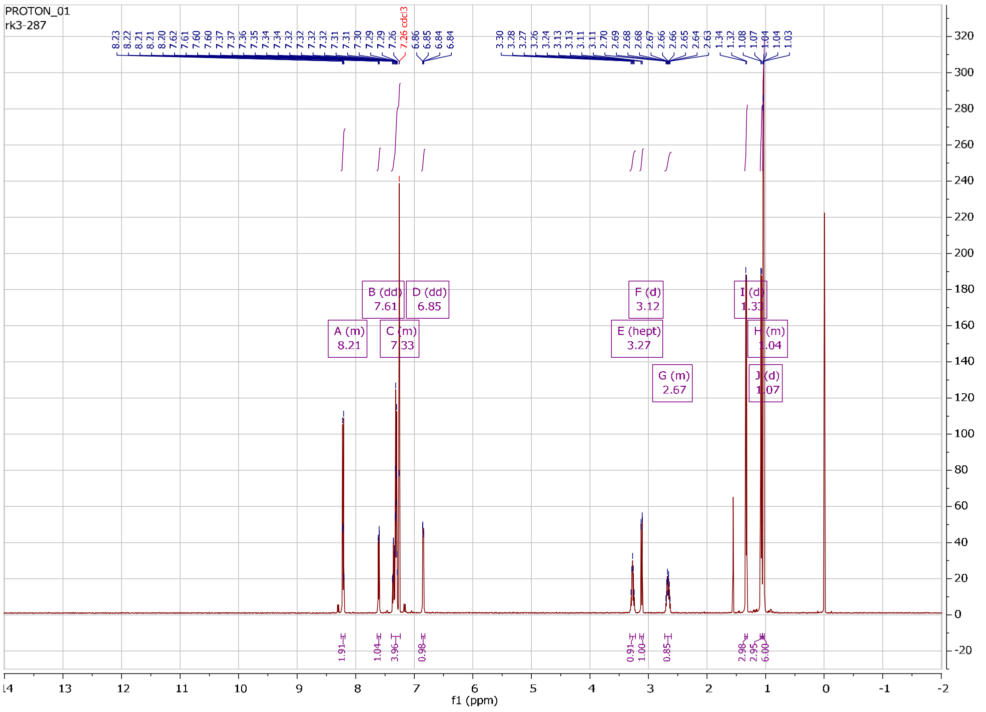

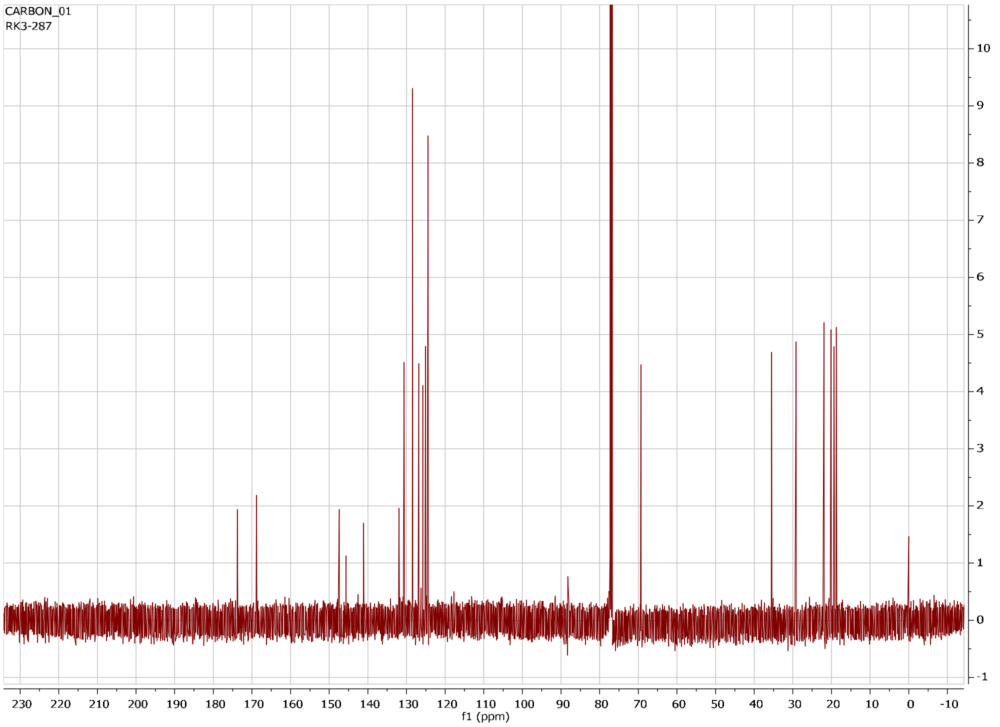

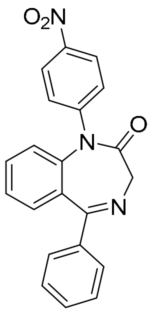

O_2N
N
O
N

3d; 82%

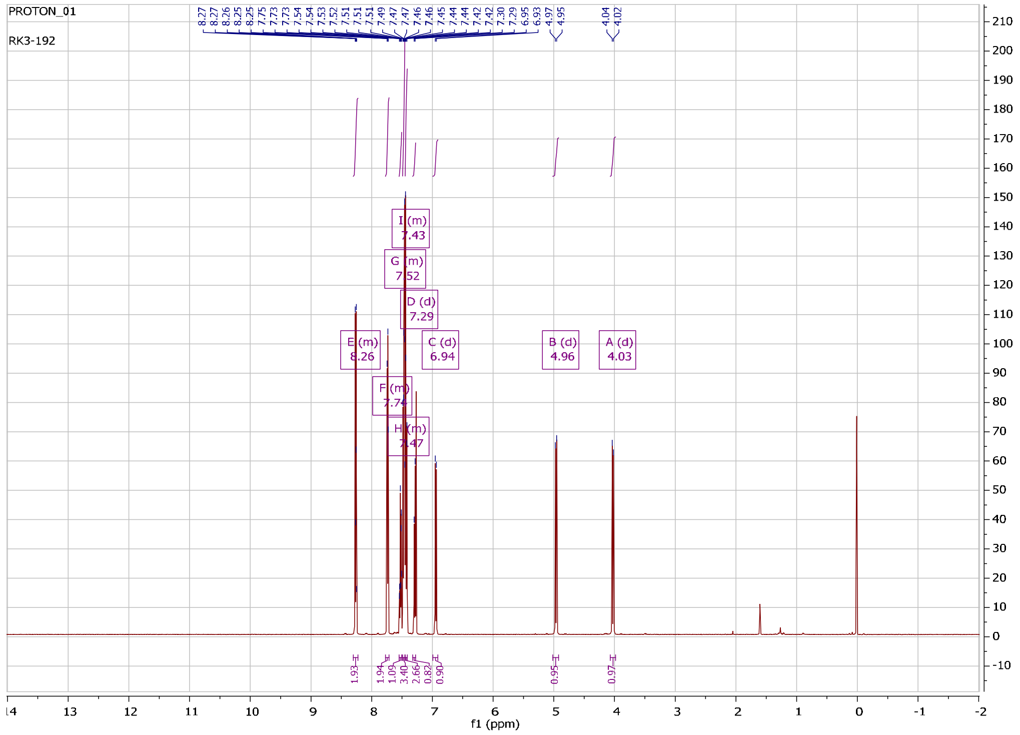

PROTON_01
RK3-192
8.27 8.27 8.26 8.25 8.25 7.75 7.73 7.73 7.54 7.54 7.53 7.52 7.51 7.51 7.51 7.49 7.47 7.47 7.46 7.46 7.45 7.44 7.44 7.42 7.42 7.30 7.29 6.95 6.93 4.97 4.95
4.04 4.02
I (m) 7.43
G (m) 7.52
D (d) 7.29
E (m) 8.26
C (d) 6.94
B (d) 4.96
A (d) 4.03
F (m) 7.74
H (m) 7.47
1.93
1.94 1.09 3.40 2.66 0.82 0.90
0.95
0.97
f1 (ppm)

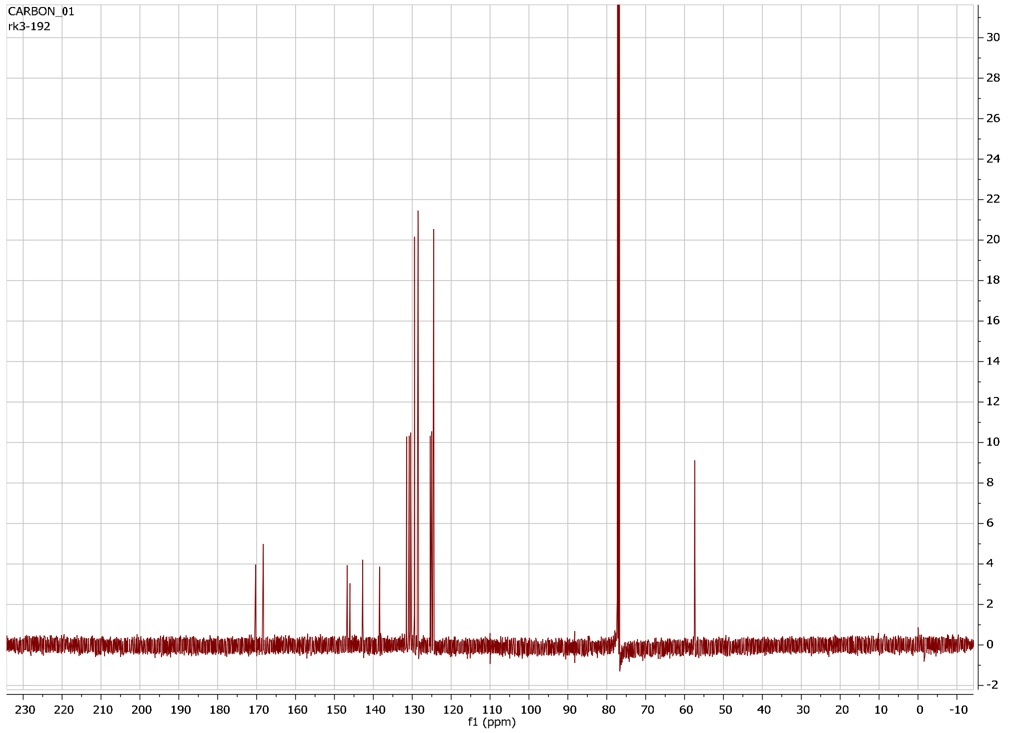

CARBON_01
rk3-192
f1 (ppm)

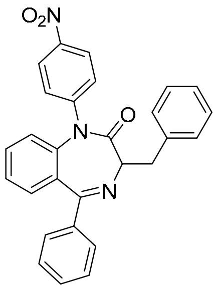

O2N
N
O
N

3e; 78%

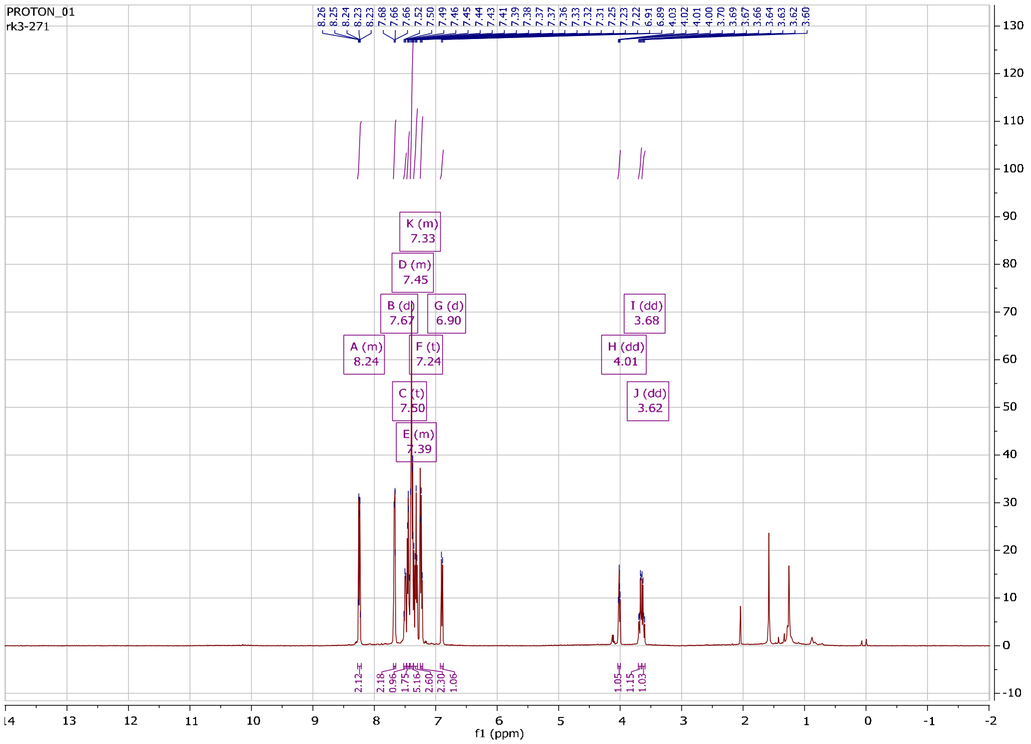

PROTON_01
rk3-271
K (m) 7.33
D (m) 7.45
B (d) 7.67
G (d) 6.90
I (dd) 3.68
A (m) 8.24
F (t) 7.24
H (dd) 4.01
C (t) 7.50
J (dd) 3.62
E (m) 7.39
f1 (ppm)

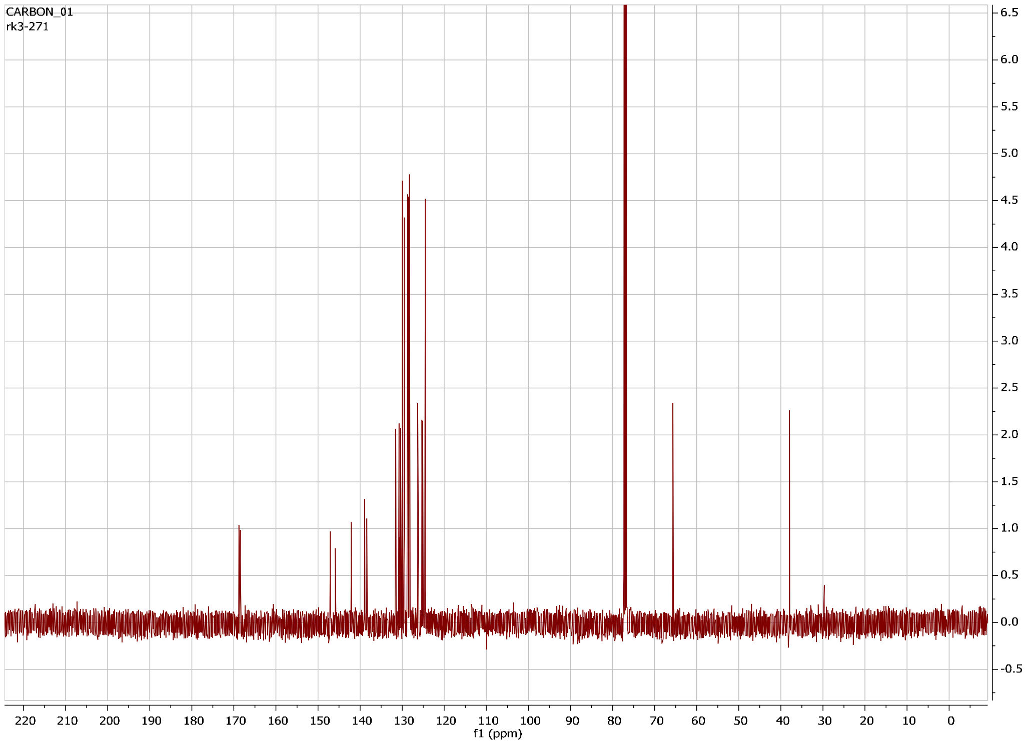

CARBON_01
rk3-271
f1 (ppm)

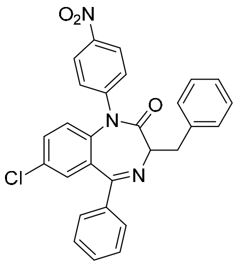

O_2N
N
O
Cl
N

3f; 75%

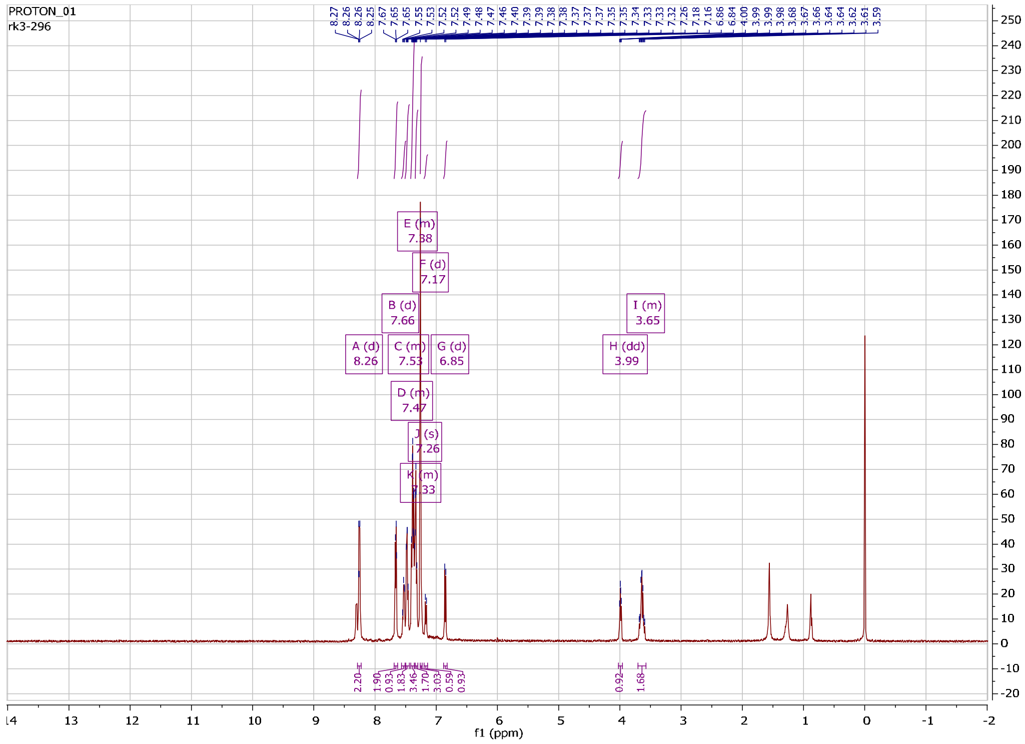

PROTON_01
rk3-296
A (d) 8.26
B (d) 7.66
C (m) 7.53
D (m) 7.47
E (m) 7.38
F (d) 7.17
G (d) 6.85
H (dd) 3.99
I (m) 3.65
J (s) 7.26
K (m) 7.33
f1 (ppm)

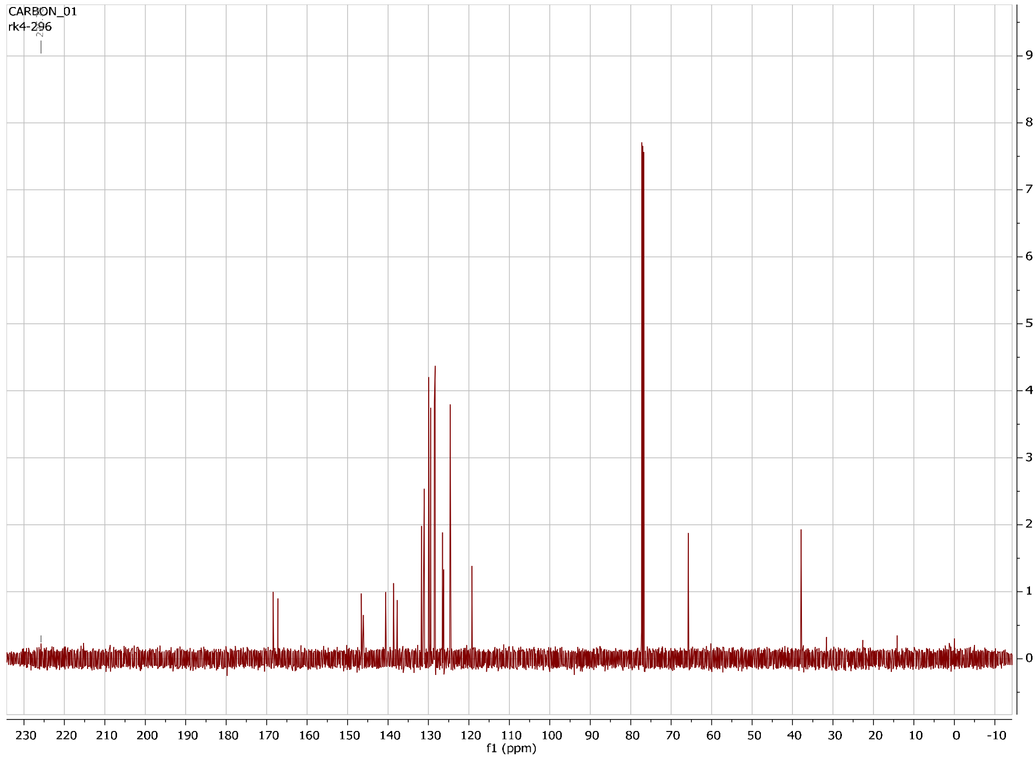

CARBON_01
rk4-296
f1 (ppm)

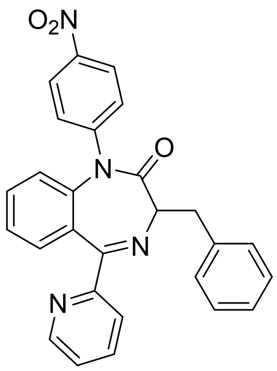
O2N
O
N
N
N

3g; 77%

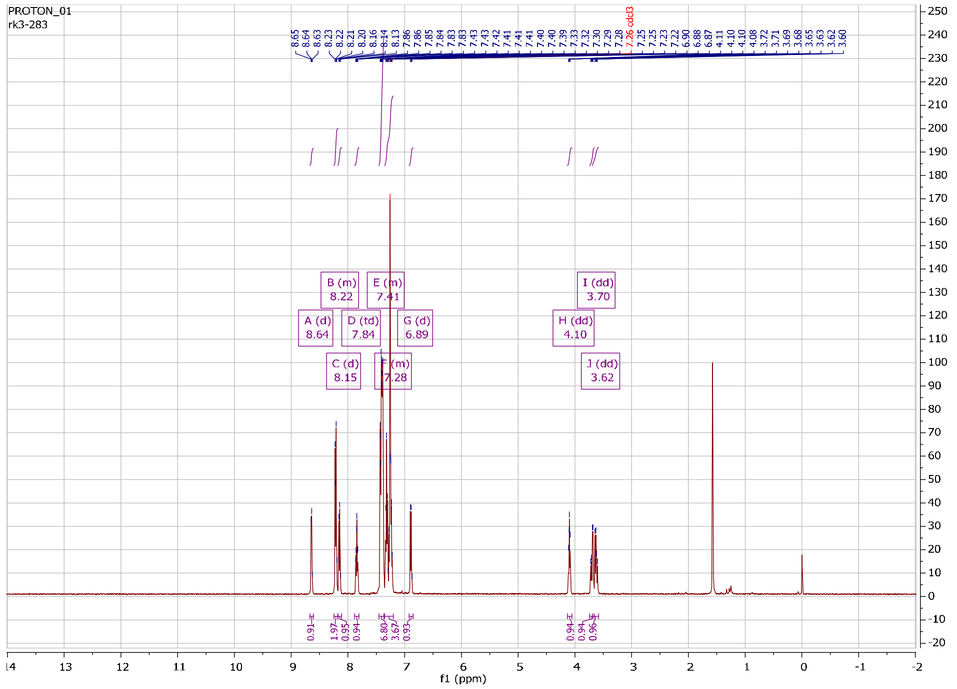
PROTON_01
rk3-283
A (d) 8.64
B (m) 8.22
C (d) 8.15
D (td) 7.84
E (m) 7.41
F (m) 7.28
G (d) 6.89
H (dd) 4.10
I (dd) 3.70
J (dd) 3.62
f1 (ppm)

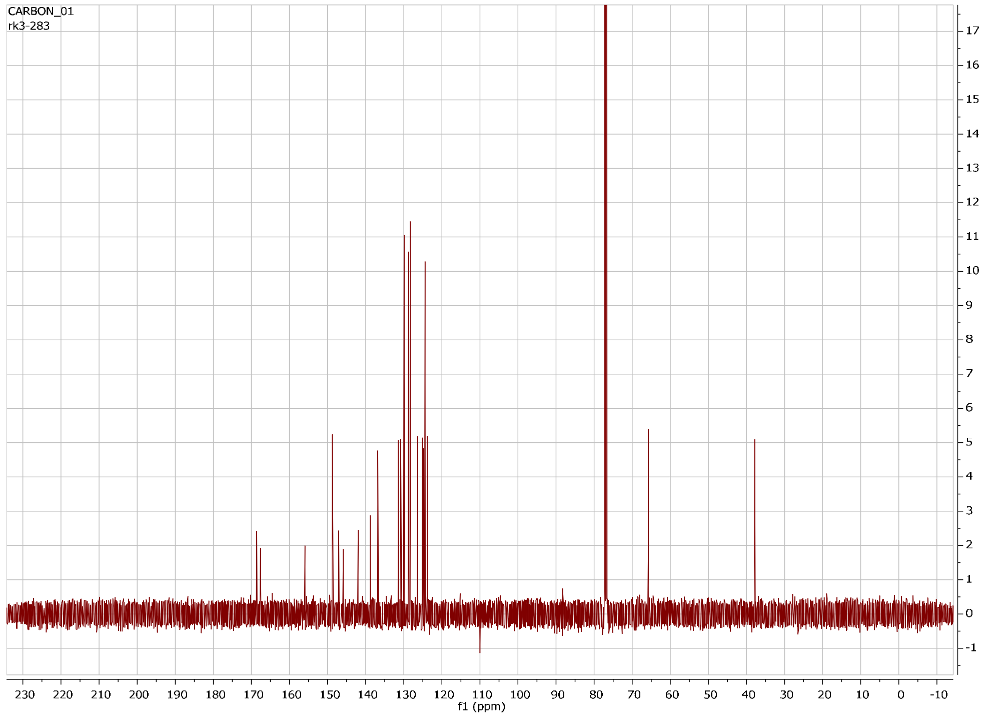
CARBON_01
rk3-283
f1 (ppm)

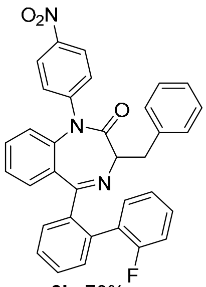

O_2N
N
O
N
F

3h; 70%

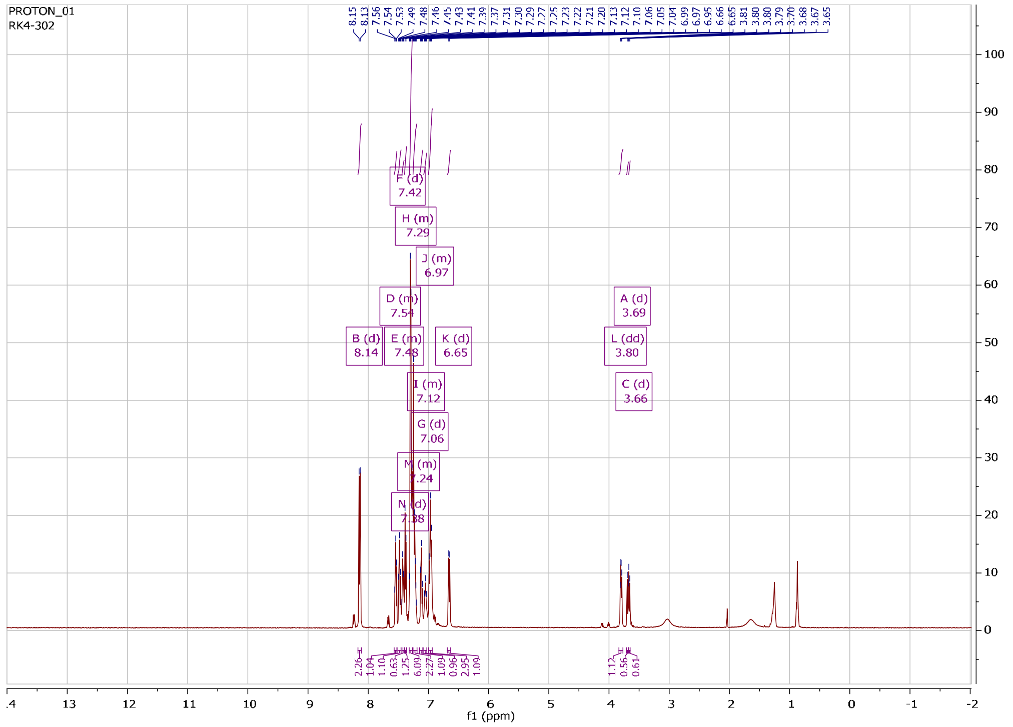

PROTON_01
RK4-302
8.15
8.13
7.56
7.54
7.53
7.49
7.48
7.46
7.45
7.43
7.41
7.39
7.37
7.31
7.30
7.29
7.27
7.25
7.23
7.22
7.21
7.20
7.13
7.12
7.10
7.06
7.05
7.04
6.99
6.97
6.95
6.66
6.65
3.81
3.80
3.80
3.79
3.70
3.68
3.67
3.65
F (d) 7.42
H (m) 7.29
J (m) 6.97
D (m) 7.54
A (d) 3.69
B (d) 8.14
E (m) 7.48
K (d) 6.65
L (dd) 3.80
I (m) 7.12
C (d) 3.66
G (d) 7.06
M (m) 7.24
N (d) 7.38
f1 (ppm)

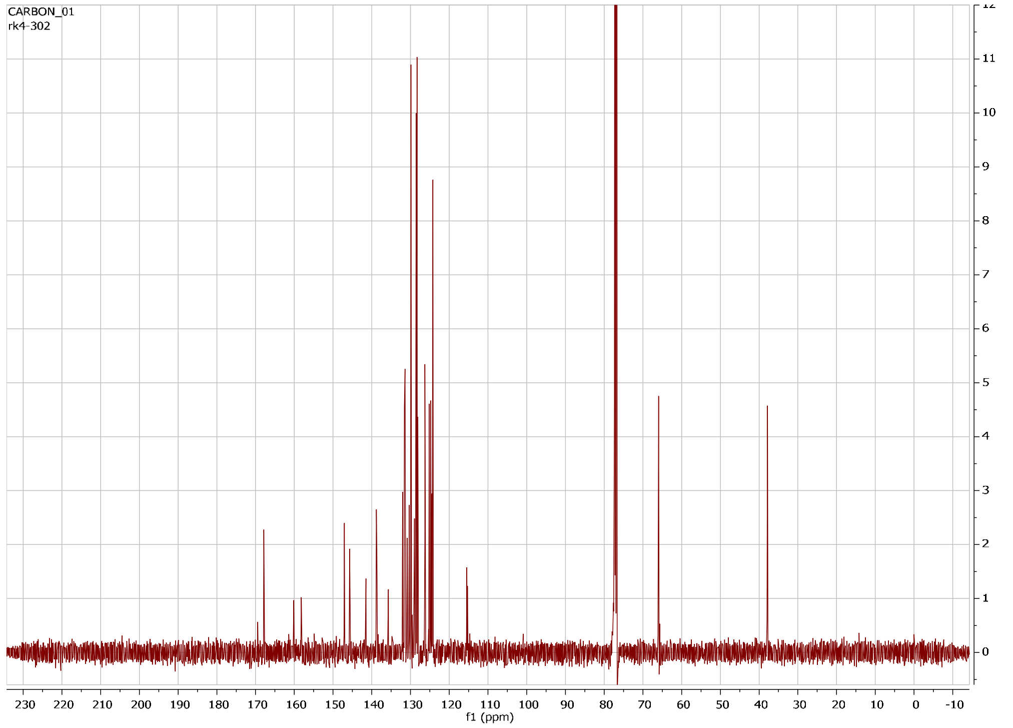

CARBON_01
rk4-302
f1 (ppm)

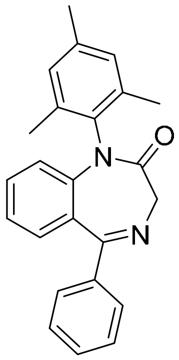

N
O
N

3i; 51%

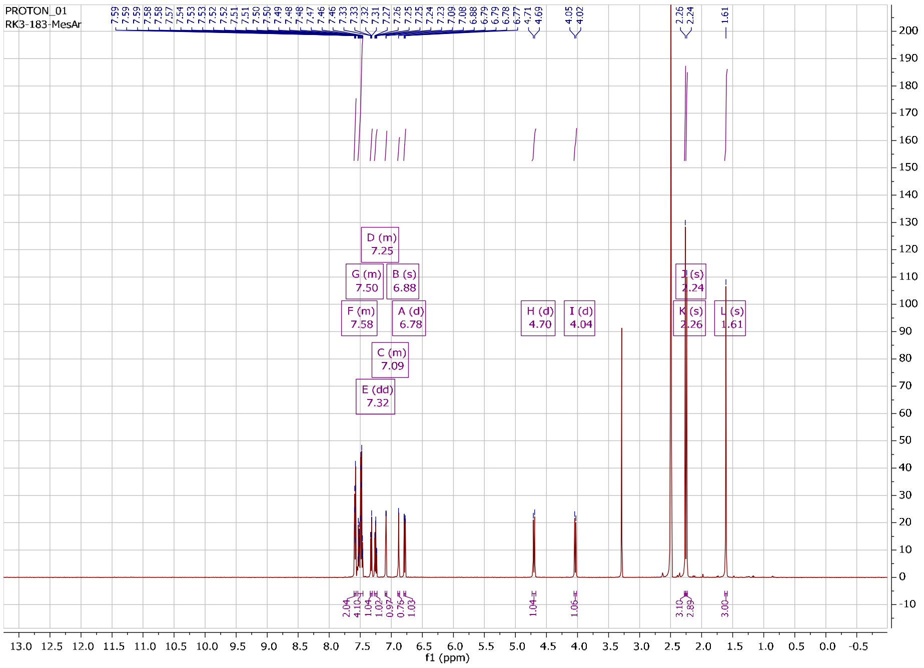

PROTON_01
RK3-183-MesAr
7.59 7.59 7.58 7.58 7.57 7.54 7.53 7.53 7.52 7.52 7.51 7.51 7.50 7.50 7.49 7.48 7.48 7.47 7.46 7.46 7.33 7.33 7.32 7.31 7.27 7.26 7.25 7.25 7.24 7.24 7.23 7.09 7.08 6.88 6.79 6.78 6.77
4.71 4.69
4.05 4.02
2.26 2.24
1.61
D (m) 7.25
G (m) 7.50
B (s) 6.88
F (m) 7.58
A (d) 6.78
C (m) 7.09
E (dd) 7.32
H (d) 4.70
I (d) 4.04
J (s) 2.24
K (s) 2.26
L (s) 1.61
2.04 4.10 1.04 1.02 0.97 0.76 1.03
1.04
1.06
3.10 2.89
3.00
f1 (ppm)

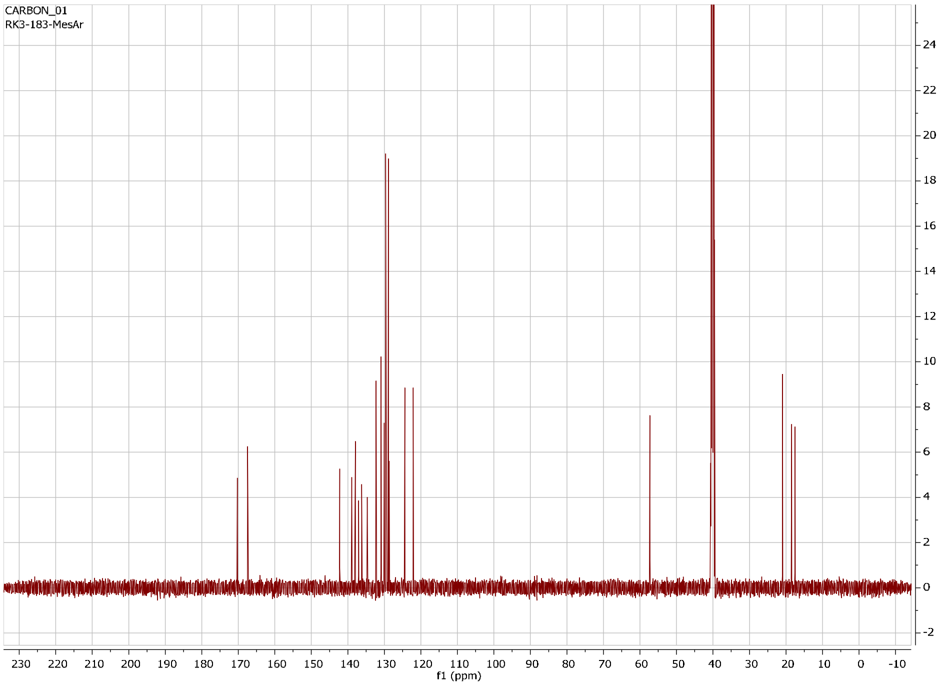

CARBON_01
RK3-183-MesAr
f1 (ppm)

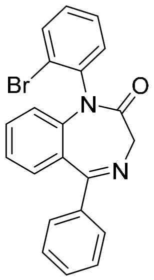
Br
N
O
N

3j; 8%

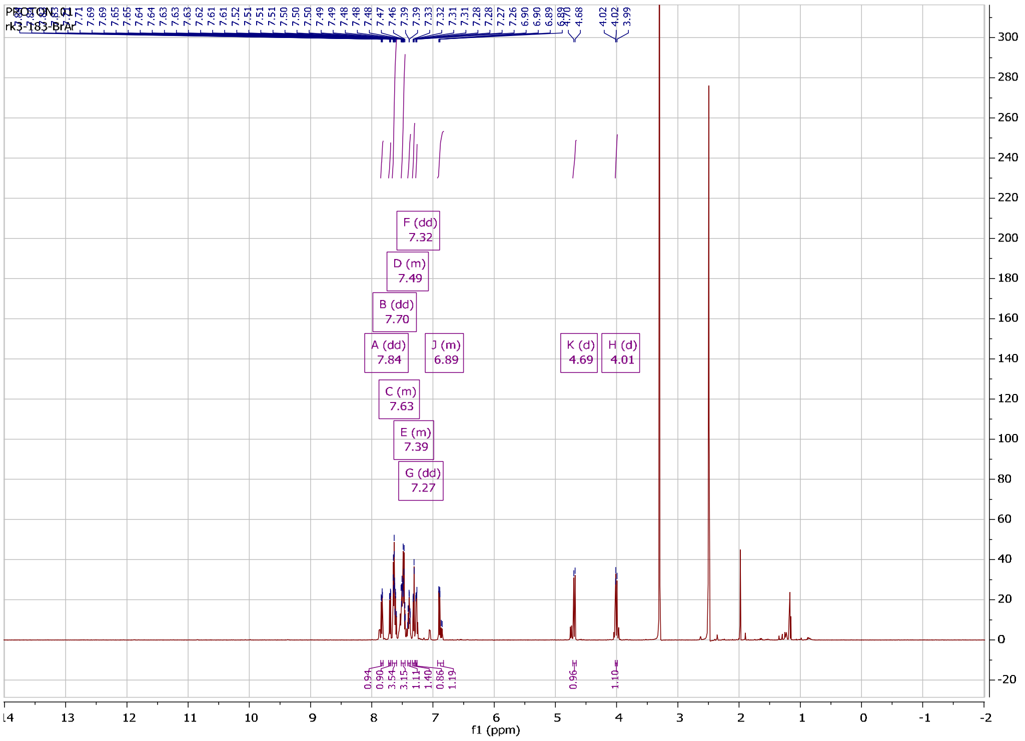
PROTON_01
rk3-183-BrAr
F (dd)
7.32
D (m)
7.49
B (dd)
7.70
A (dd)
7.84
J (m)
6.89
K (d)
4.69
H (d)
4.01
C (m)
7.63
E (m)
7.39
G (dd)
7.27
f1 (ppm)

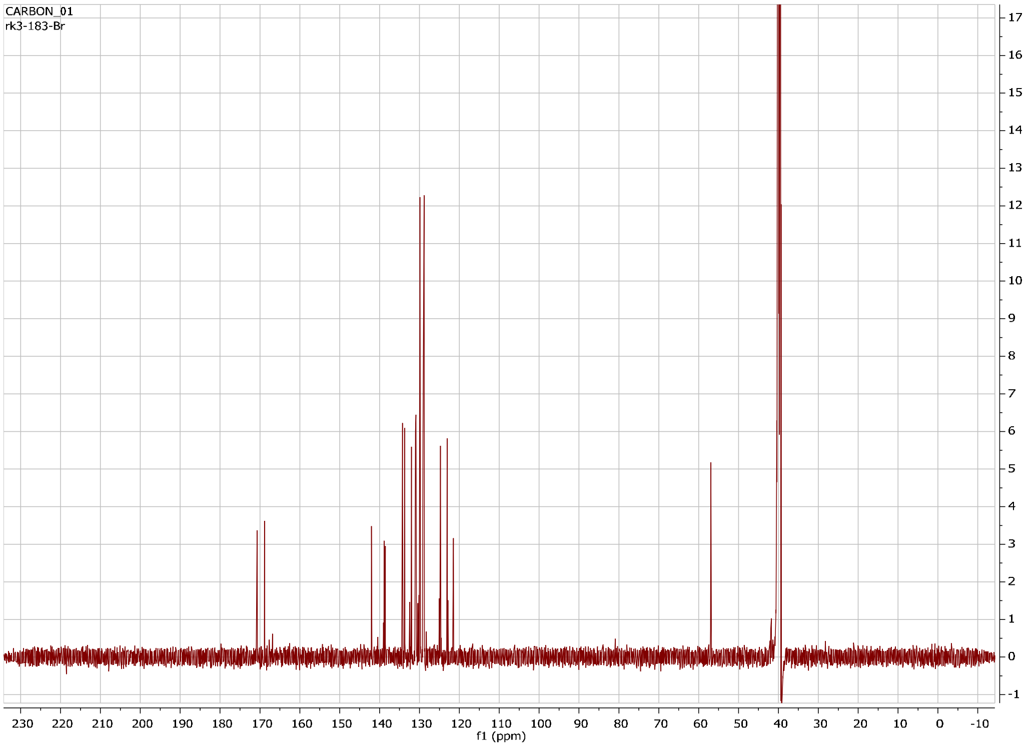
CARBON_01
rk3-183-Br
f1 (ppm)

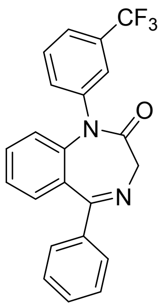

CF3
N
O
N

3k; 42%

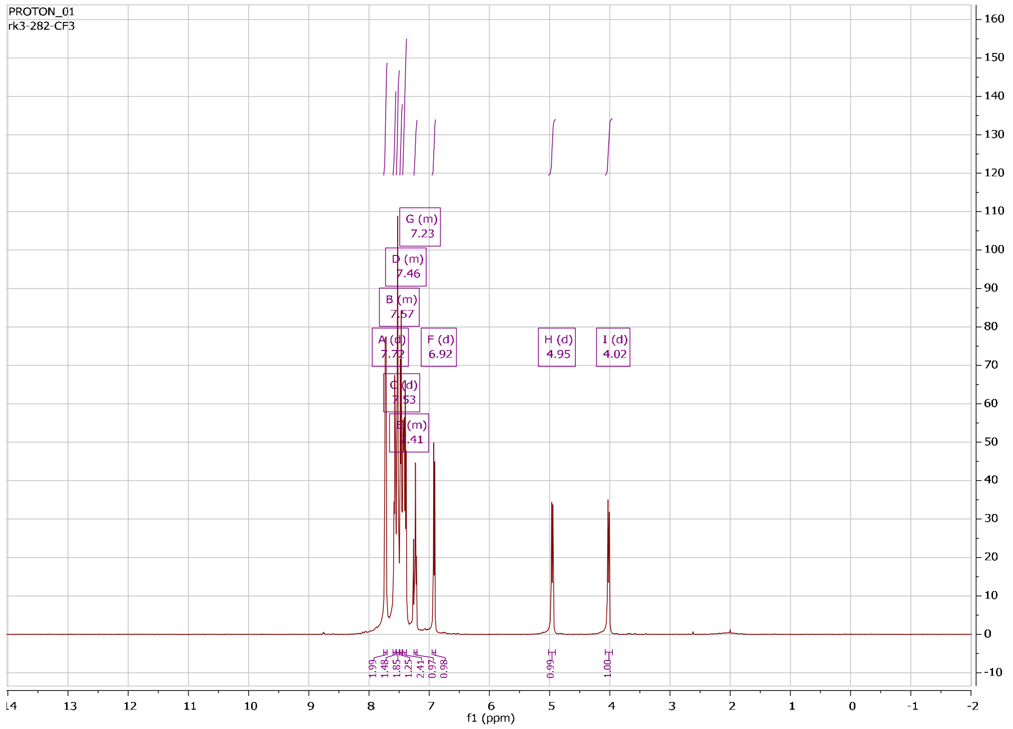

PROTON_01
rk3-282-CF3
G (m)
7.23
D (m)
7.46
B (m)
7.57
A (d)
7.72
F (d)
6.92
H (d)
4.95
I (d)
4.02
C (d)
7.53
E (m)
7.41
1.99
1.48
1.85
1.25
2.41
0.97
0.98
0.99
1.00
f1 (ppm)

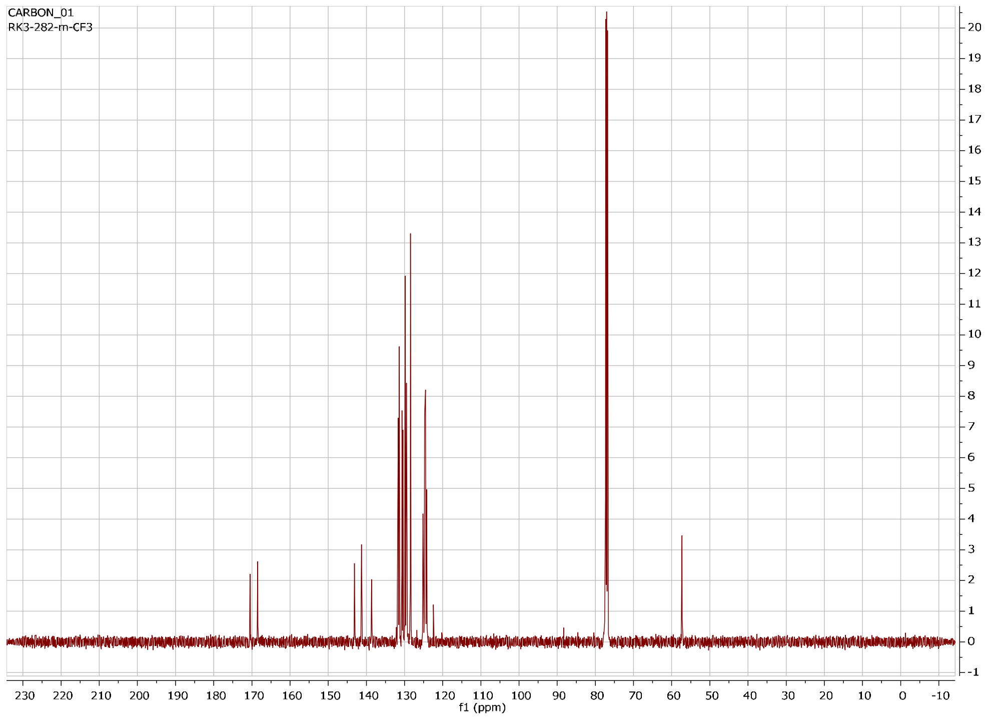

CARBON_01
RK3-282-m-CF3
f1 (ppm)

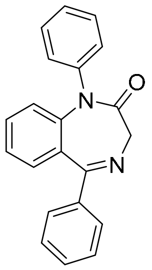

3l; 9%

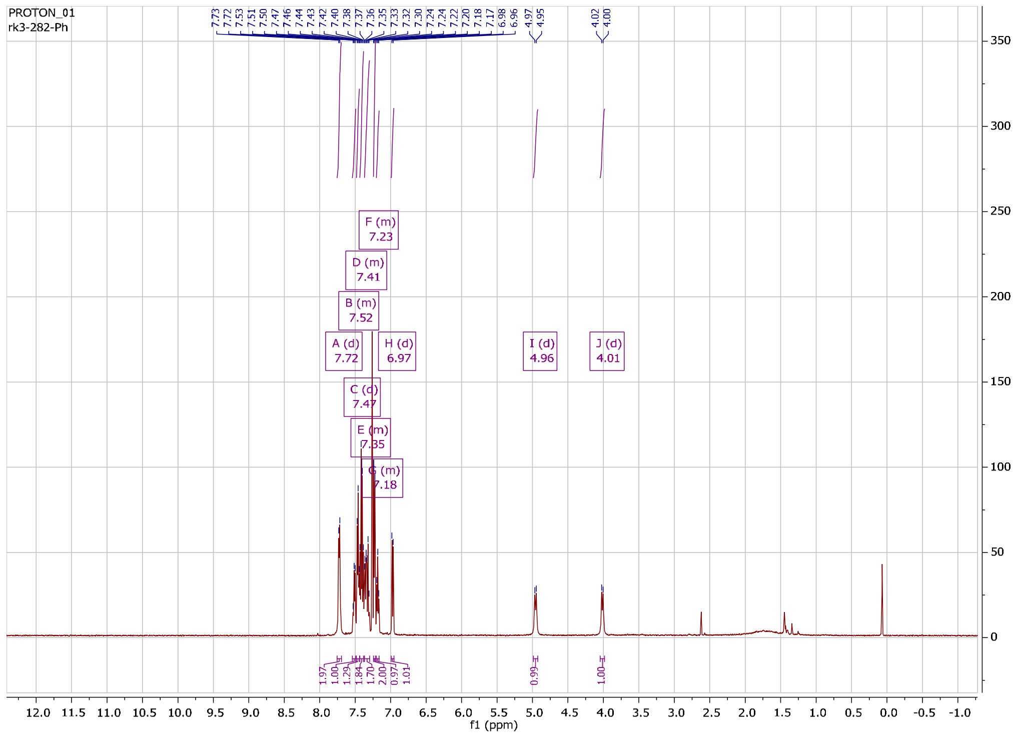
PROTON_01
rk3-282-Ph
A (d) 7.72
B (m) 7.52
C (d) 7.47
D (m) 7.41
E (m) 7.35
F (m) 7.23
G (m) 7.18
H (d) 6.97
I (d) 4.96
J (d) 4.01
f1 (ppm)

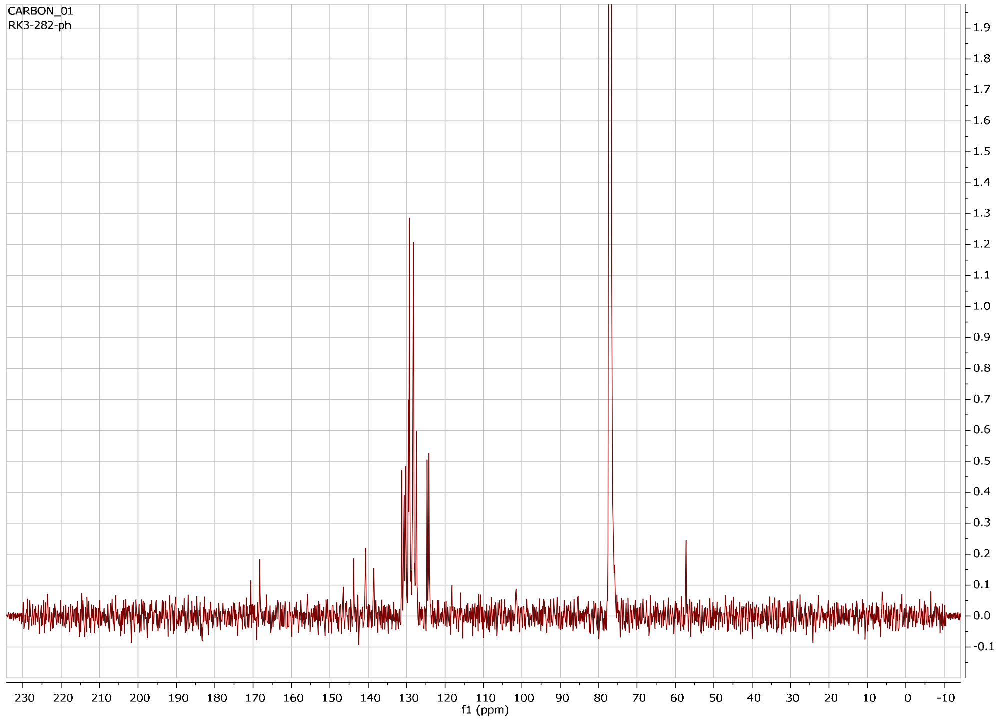
CARBON_01
RK3-282-ph
f1 (ppm)

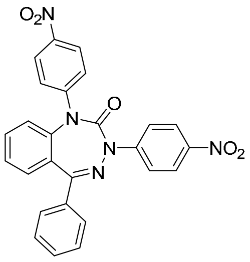
O2N
O
N
N
N
NO2

7; 51%

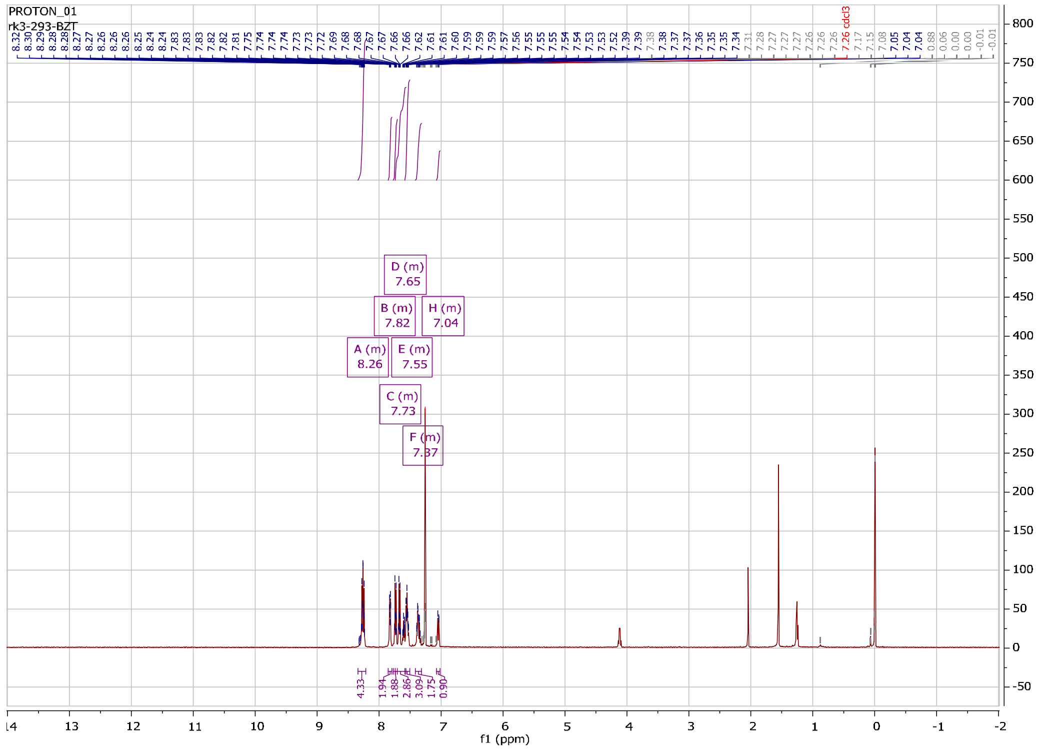
PROTON_01
rk3-293-BZT
A (m)
8.26
B (m)
7.82
C (m)
7.73
D (m)
7.65
E (m)
7.55
F (m)
7.37
H (m)
7.04
4.33
1.91
1.88
2.86
3.09
1.75
0.90
f1 (ppm)

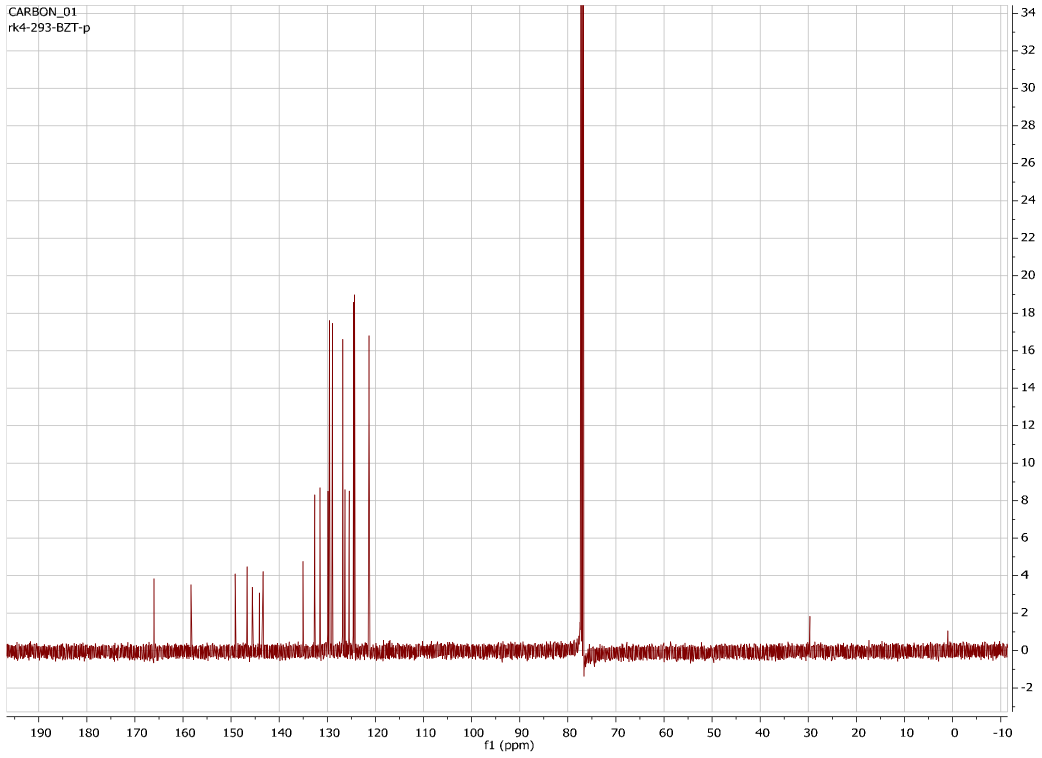
CARBON_01
rk4-293-BZT-p
f1 (ppm)

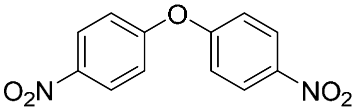
O
O_2N
NO_2

10; 43%

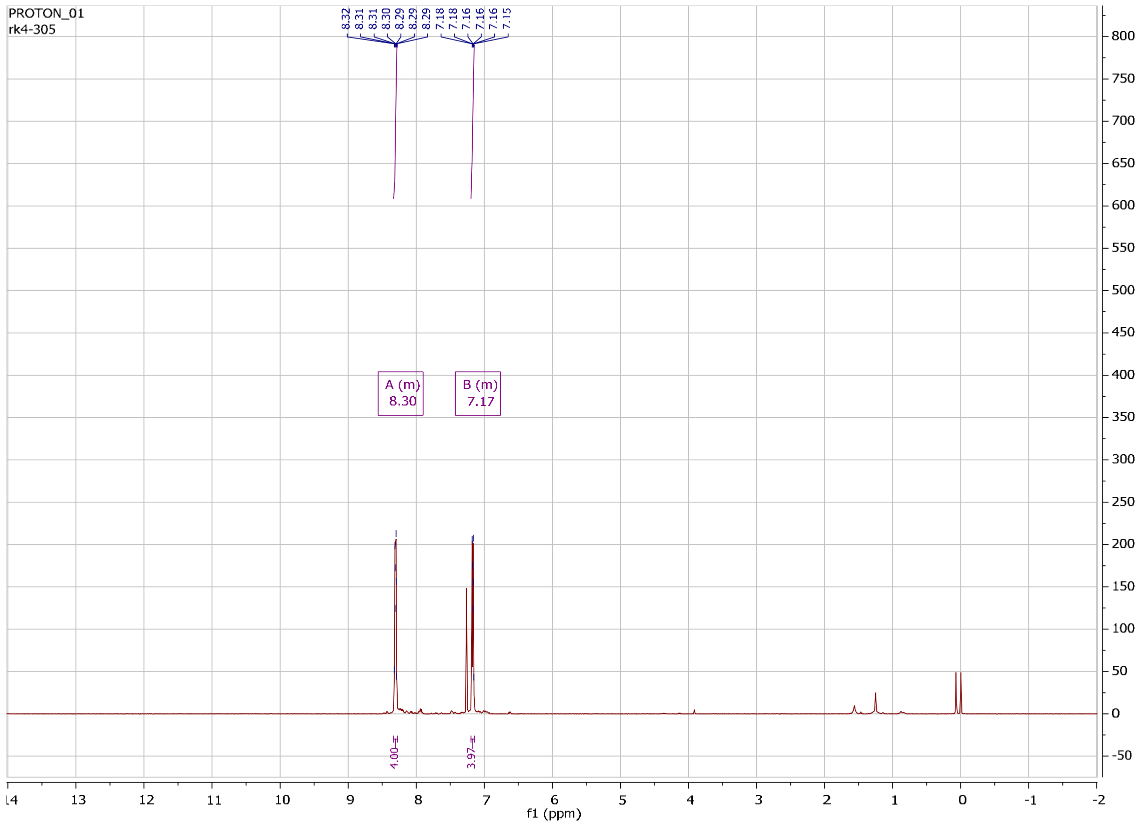
PROTON_01
rk4-305
8.32
8.31
8.31
8.30
8.29
8.29
8.29
7.18
7.18
7.16
7.16
7.16
7.15
A (m)
8.30
B (m)
7.17
4.00
3.97
f1 (ppm)

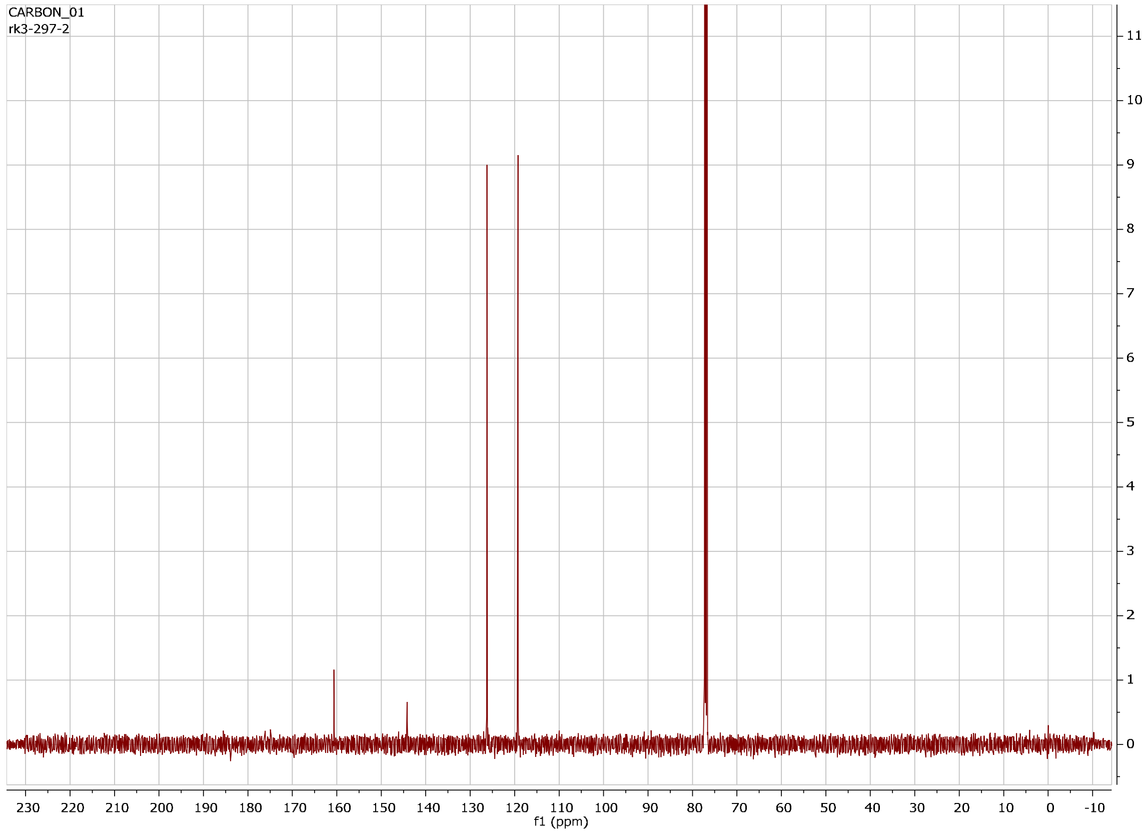
CARBON_01
rk3-297-2
f1 (ppm)
